# Integrating Imaging and Circulating Tumor DNA Features for Predicting Patient Outcomes

**DOI:** 10.3390/cancers16101879

**Published:** 2024-05-15

**Authors:** Mark Jesus M. Magbanua, Wen Li, Laura J. van ’t Veer

**Affiliations:** 1 Department of Laboratory Medicine, University of California San Francisco, San Francisco, CA 94115, USA; laura.vantveer@ucsf.edu; 2 Department of Radiology and Biomedical Imaging, University of California San Francisco, San Francisco, CA 94115, USA; wen.li@ucsf.edu

**Keywords:** liquid biopsy, circulating tumor DNA (ctDNA), imaging, models

## Abstract

**Simple Summary:**

Predicting which patients will respond to therapy or experience disease relapse can help clinicians select treatments that could slow down or prevent the spread of cancer. Clinicians have routinely used imaging to measure the size of the tumor to assess whether or not it is responding to treatment. A recent development for monitoring tumors involves a test to detect mutated DNA shed by tumors into the blood, called circulating tumor DNA (ctDNA). The authors searched the scientific literature to find studies that have combined imaging and ctDNA to build tools that can predict treatment response or patient survival. The authors noted that only a few studies have been reported, indicating that this field is new and needs further exploration. These early studies, however, showed that combining these two clinical tests (imaging + ctDNA) may improve the prediction of tumors’ response to therapy and the return of cancer. While promising, these tools need to be refined to improve the accuracy of the predictions and the results confirmed in more extensive studies.

**Abstract:**

Biomarkers for evaluating tumor response to therapy and estimating the risk of disease relapse represent tremendous areas of clinical need. To evaluate treatment efficacy, tumor response is routinely assessed using different imaging modalities like positron emission tomography/computed tomography or magnetic resonance imaging. More recently, the development of circulating tumor DNA detection assays has provided a minimally invasive approach to evaluate tumor response and prognosis through a blood test (liquid biopsy). Integrating imaging- and circulating tumor DNA-based biomarkers may lead to improvements in the prediction of patient outcomes. For this mini-review, we searched the scientific literature to find original articles that combined quantitative imaging and circulating tumor DNA biomarkers to build prediction models. Seven studies reported building prognostic models to predict distant recurrence-free, progression-free, or overall survival. Three discussed building models to predict treatment response using tumor volume, pathologic complete response, or objective response as endpoints. The limited number of articles and the modest cohort sizes reported in these studies attest to the infancy of this field of study. Nonetheless, these studies demonstrate the feasibility of developing multivariable response-predictive and prognostic models using regression and machine learning approaches. Larger studies are warranted to facilitate the building of highly accurate response-predictive and prognostic models that are generalizable to other datasets and clinical settings.

## 1. Introduction

Predictive and prognostic biomarkers can help guide treatment to improve the out comes of patients with cancer [1]. Biomarkers that predict response to treatment can aid therapeutic decisions to prevent or delay disease relapse [2]. For instance, patients whose tumors are predicted to respond poorly to a specific therapy may benefit from an early switch in treatment to improve the chances of achieving a favorable response. Additionally, prognostic biomarkers that can predict disease relapse after the surgical resection of primary cancer can help guide therapy in the adjuvant setting to prevent metastatic recurrence [3]. In patients with metastatic disease, biomarkers can aid in selecting efficacious agents that could delay disease progression [4]. Thus, using biomarkers to inform treatment decisions can lead to higher response rates, reduced exposure to toxicities of ineffective or unnecessary treatments, and improved patient survival.

Biomedical imaging plays a crucial role in cancer treatment, from planning therapy [5] to real-time monitoring and response assessment [6,7,8]. Imaging biomarkers derived from magnetic resonance imaging (MRI), positron emission tomography (PET), or computed tomography (CT) have extensively been used in the assessment of tumor response to cancer treatment [8]. Tumor burden measurements from imaging, such as longest diameter or tumor volume, have also shown promise in predicting patient outcomes and guiding treatment decisions in the clinic [9,10]. Recent advances in machine learning/deep learning and radiomics have allowed a more extensive and iterative search for imaging features and model parameters that are highly predictive of patient outcomes [11]. 

More recently, liquid biopsy biomarkers from blood [12], e.g., circulating tumor DNA (ctDNA), have been developed to assess treatment response and estimate the risk of recurrence and disease progression [13]. ctDNA-based biomarkers represent less invasive approaches to evaluating tumor response and patient survival and, therefore, can be measured repeatedly via serial blood draws to improve prediction accuracy. In addition, ctDNA can be used to monitor treatment response or resistance with minimal risks to the patient [14,15].

In this mini-review, we reviewed studies that combined ctDNA and imaging biomarkers to build models for predicting patient outcomes across different cancer types and therapeutic settings. We described the different platforms used for imaging and ctDNA analysis. We then examined the biomarkers and approaches the investigators utilized to create models for predicting treatment response and survival. Finally, we summarized the key findings of each study and their implications for improving cancer treatment.

## 2. Materials and Methods

### 2.1. Literature Search

**Figure 1:**
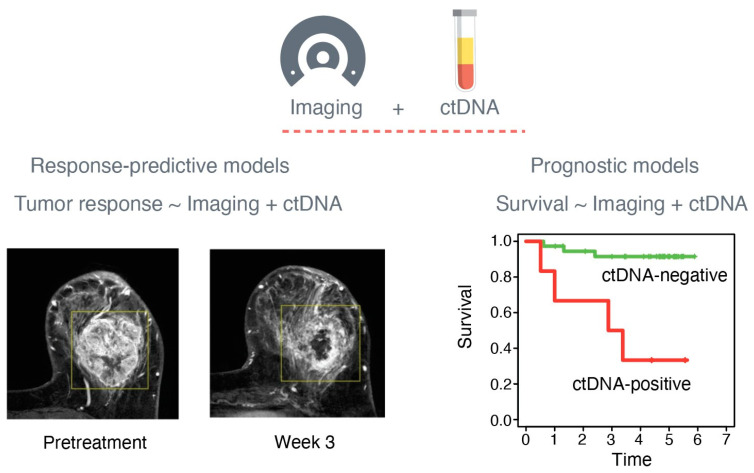
Imaging and circulating tumor DNA (ctDNA) biomarkers for predicting patient outcomes. Quantitative biomarkers from imaging and ctDNA can be combined to build response-predictive and prognostic models to predict tumor response and survival, respectively. Images of the breast by magnetic resonance imaging were adapted from Li et al. [16].

We searched the scientific literature to identify studies that leveraged both imaging and ctDNA data to build integrative predictive and prognostic models (Figure 1). We used the keywords “imaging” and “ctDNA” in a PubMed search and found 496 entries. After reviewing the article titles, 36 publications were deemed candidates for a full abstract review. Review articles and original studies that only reported correlations between imaging and ctDNA biomarkers without modeling were excluded from further review.

### 2.2. ctDNA Detection Platforms and Biomarkers

One of the most well-studied blood-based liquid biopsy biomarkers is ctDNA [17]. In most cases, this biomarker represents a minute fraction of the total cell-free DNA floating in the plasma and is exclusively shed into circulation by tumor cells [12]. There are many platforms for detecting ctDNA, which are relatively rare, small fragments of DNA molecules present in a cell-free DNA background, mainly derived from dying hematopoietic cells [17]. ctDNA detection platforms can be classified into two major categories: tumor agnostic and tumor informed [18]. Tumor-agnostic platforms do not require sequence information of tumors of origin but are based on detecting altered copies of commonly mutated cancer-related genes such as *TP53* and *PIK3CA*. Mutant and wild-type copies of the genes are sequestered from plasma by hybridization capture. Then, the captured DNA molecules are subjected to next-generation sequencing (NGS) to estimate the variant allele fraction (VAF, also called mutant allele frequency or MAF) or mutant copies per mL of plasma. In contrast, tumor-informed platforms require the sequencing of the original tumor (e.g., whole-exome sequencing), and a panel of patient-specific mutations is selected. The region containing the mutation in each gene is then amplified by polymerase chain reaction (PCR), and the amplicons are subjected to deep NGS to detect mutated copies.

### 2.3. Imaging Platforms and Quantitative Biomarkers

Biomedical imaging consists of many platforms for non-invasive quantitative measurements of tumors from medical images. The most common modalities used in conjunction with cancer treatment are dynamic contrast-enhanced MRI, CT, X-ray, and PET. The most frequently used quantitative imaging biomarkers from the first three modalities include measures of tumor size, e.g., longest diameter and tumor volume [19]. Different from MRI, CT, and X-ray, which represent anatomical imaging modalities, PET and PET/CT are functional imaging modalities measuring the metabolic or biochemical function of tissues or organs [20,21]. In particular, fluorine isotope 18 (^18^F)-fluorodeoxyglucose (FDG) PET can be used to measure FDG uptake in targeted organs such as the liver, brain, or breast. The most common measurement derived from FDG PET imaging is the standardized uptake value (SUV, unit: grams per milliliter) reflecting the relative uptake of FDG in an organ normalized to the injected dose of FDG and patient body weight [22]. The maximum SUV (SUVmax) is the tumor’s maximum FDG uptake value measured within a defined region of interest (ROI). The measurement of SUVmax has been demonstrated to be highly reproducible despite variability in the ROI delineation by image readers [23,24]. Metabolic tumor volume (MTV) is the volume of metabolically active tumor cells that have uptake above the pre-defined threshold on FDG PET examination, while the total lesion glycolysis (TLG) is the product of the mean SUV and the MTV within the ROI [25].

### 2.4. Response and Survival Endpoint

Outcomes refer to the results of the treatment and care the patient receives in a clinical setting. Endpoints are specific outcome measures and can refer to whether a patient’s tumor responds to treatment or not (response endpoint) or how long the patient lives with or without disease relapse (survival endpoint). The response endpoints used in the studies discussed in this review include pathologic complete response (pathCR) and objective response, as defined by the Response Evaluation Criteria in Solid Tumors (RECIST) guidelines, and the survival endpoints include overall survival (OS), progression-free survival (PFS), and distant recurrence-free survival (DRFS).

PathCR is defined as the absence of invasive cancer in the primary tumor bed following neoadjuvant therapy (treatment administered before surgery). PathCR, assessed at surgery, allows for the rapid evaluation of drug efficacy and has been proposed as a surrogate endpoint of long-term clinical benefit [26]. Studies showed that achieving a pathCR is a strong predictor of favorable survival [27,28]. Objective response is based on RECIST guidelines, which provide a framework for tumor response evaluation based on quantitative changes in tumor burden assessed by imaging or clinical exams. RECIST was initially published in 2000 [29] and updated in 2009 (RECIST 1.1 [30]). Imaging modalities often used for objective response evaluation include MRI, CT, X-ray, and FDG PET. Four types of objective responses can be measured at the target lesions [31]: (1) complete response (CR) is defined as the disappearance of all lesions and pathologic lymph nodes; (2) partial response (PR) refers to a ≥30% decrease in the sum of the longest diameters of the target lesions; (3) stable disease (SD) is neither PR nor (4) progressive disease (PD), defined as an increase of at least 20% in the sum of the longest diameters of target lesions with an absolute increase of ≥5 mm, or the development of one or more new lesions [30].

OS is usually defined as the time interval between the start of treatment and death from any cause and is considered the “gold standard” in assessing treatment efficacy [32]. An early surrogate endpoint for OS is PFS, the time interval between the start of treatment and the documentation of disease progression or death from any cause [33]. In clinical studies involving patients with early stage (non-metastatic) cancer, the endpoint often used is DRFS, defined as the time interval between treatment start and the diagnosis of distant recurrence or death from any cause [27].

## 3. Results

In total, seven original studies were chosen for a full review, including six from the literature search [34,35,36,37,38,39]. One additional article was found using a separate search engine, as the authors used the term “plasma tumor DNA (ptDNA)” instead of ctDNA [40], and thus, the article was missed during the initial PubMed search.

The studies involved patients with different cancer types, including three non-small cell lung carcinoma (NSCLC), one high-grade serous ovarian cancer (HGSOC), one prostate, and two breast cancer studies (Table 1). Two studies were performed in early stage (Stage I–III), two in advanced (Stage III–IV), and three in metastatic (Stage IV) cancer settings. Patients received various types of treatment, including chemotherapy and targeted therapies. Two studies used machine learning approaches to identify ctDNA and imaging features that are predictive of patient outcomes. Detailed information regarding the studies is summarized in Table 2 and Table 3.

### 3.1. Combined Imaging and ctDNA Biomarkers for Predicting Survival

This section discusses two studies on NSCLC [36,39] and one on breast cancer [38] that built prognostic models combining imaging and ctDNA biomarkers. 

Yousefi and colleagues investigated whether imaging features of tumors from patients with metastatic NSCLC can be combined with ctDNA data to improve the prediction of survival after epidermal growth factor receptor (EGFR)-targeted therapy [36]. The investigators extracted 429 imaging features of primary tumors from 40 patients using data from pretreatment chest CT scans. These features were then used in unsupervised hierarchical clustering to group tumors into two imaging phenotypes. NGS of cell-free DNA (cfDNA) was used to estimate the number of ctDNA mutations in the blood. In addition to imaging and ctDNA information, Yousefi et al. used clinical data, including age, smoking status, and ECOG (Eastern Cooperative Oncology Group) performance status, to calculate a prognostic score for each patient. Using the median value of this measure, the patients were divided into two groups. Survival analysis showed that using the prognostic score derived from ctDNA, clinical variables, and imaging phenotypes improved the prediction of PFS and OS.

Ottestad et al. examined whether ctDNA levels are correlated with tumor metabolic activity as measured by PET/CT [39]. ctDNA data from 63 patients with stage I–III NSCLC were obtained from previously published studies [41,42,43]. A patient-specific NGS panel or digital droplet polymerase chain reaction (ddPCR) was used to detect ctDNA, and ctDNA levels were reported as VAF. The levels of ctDNA were positively correlated with PET/CT features, including MTV and TLG, but not with SUVmax. Univariable Cox proportional hazard analysis showed that ctDNA detection, MTV, TLG, and SUVmax were significantly associated with PFS and OS. In a multivariable Cox model, none of the predictors were significantly associated with PFS, while only ctDNA remained a significant prognostic factor for OS.

Everolimus, an mTOR inhibitor, when combined with endocrine therapy, has been shown to improve PFS in patients with breast cancer [44]. Exemestane, a steroidal (Type I) endocrine therapy, inactivates aromatase, a key enzyme in estrogen biosynthesis. Gombos and colleagues reported the results of a trial (Pearl) that assessed response by PET/CT and ctDNA 14 days after everolimus–exemestane treatment initiation [38]. CfDNA from plasma was subjected to deep NGS to detect mutations in 40 cancer-specific genes. The investigators examined whether a prognostic model including ctDNA and imaging features can predict PFS and identify patients who do not benefit from adding everolimus to exemestane. Using a “post-hoc” cut-off of <15% for SUVmax decrease to dichotomize patients into non-responders and responders, they found a significant difference in PFS between the two groups. ctDNA on treatment day 14 was also shown to be significantly associated with PFS. Multivariate Cox proportional hazard analysis showed that the detection of ctDNA and the absence of PET/CT response on day 14 identified patients with a low probability of benefiting from everolimus–exemestane treatment.

### 3.2. Integrating Imaging and ctDNA Biomarkers in Predictive and Prognostic Models

Next, we highlight two studies integrating imaging and ctDNA biomarkers to build models for predicting response and survival [35,37].

The first study involves patients with stage III–IV NSCLC receiving chemotherapy [37]. Fiala et al. examined the prognostic value of PET/CT and ctDNA measurements collected at pretreatment and after two cycles of chemotherapy (follow up) in 84 patients. The PCR/DCE-based heteroduplex method (heteroduplex analysis after the amplification of the mutated tumor-specific gene fragment) was used to detect mutations in genes commonly altered in lung cancer, e.g., *EGFR*, *KRAS*, *TP53*, *PIK3CA*, and *BRAF*. VAF was used as the quantitative ctDNA measure for correlation analyses. The authors found that pretreatment and ∆ctDNA VAF values were significantly correlated with PET/CT features, MTV, TLG, and IC. They also observed that follow-up ctDNA and changes in all PET/CT parameters were associated with treatment response based on RECIST guidelines. Receiver-operating characteristic (ROC) analyses showed that adding follow-up ctDNA to ∆SUVmax (%) improved the prediction of objective response and PFS.

Second, our group demonstrated the feasibility of building models to predict pathCR and DRFS in patients with high-risk, early stage (Stage II–III) breast cancer receiving neoadjuvant chemotherapy (NAC) in the I-SPY2 trial [35]. The trial used MRI for serial imaging of the breast to assess tumor burden, reported as FTV. ctDNA was analyzed using a tumor-informed test (SignateraTM) involving the sequencing of pretreatment tumors and the selection of up to 16 clonal mutations (high VAF in tissue). PCR primers were then designed to amplify the regions containing the alterations, and the amplicons were subjected to deep NGS to detect mutant copies (ctDNA) in cfDNA. ctDNA was reported as a dichotomous (positive vs. negative) and continuous variable (MTM/mL). ROC analysis showed that adding ctDNA to the response-predictive model containing FTV only improved the early prediction of pathCR (3 weeks after treatment initiation). A bivariable Cox proportional hazard analysis showed that the ctDNA detection and larger FTV after NAC were significantly associated with DRFS.

### 3.3. Machine Learning Approaches to Discover Predictive and Prognostic Imaging and ctDNA Biomarkers

Machine learning is a novel approach to finding features predictive of an outcome, e.g., response vs. no response or recurrence vs. no recurrence. First, the data are split into training and test sets. The training set (usually a larger portion of the complete dataset, e.g., 70–80%) is then used to build a prediction model by learning which features are associated with an outcome. The model’s performance is then evaluated by applying the parameters of the model, learned in the training set, to a test set, the remaining dataset that the model has not seen. Accurate predictions in the test set and, sometimes, in an external validation set indicate the robustness of the model (generalizability). This section discusses two studies that used machine learning to find imaging and ctDNA features predictive of treatment response [34] or survival [40].

In the first study, Conteduca et al. investigated whether pretreatment ctDNA levels reflect metabolic tumor burden assessed by PET/CT and better predict survival in combination with imaging features [40]. CtDNA was detected using targeted NGS in 102 patients with Stage IV castration-resistant prostate cancer treated with abiraterone, an inhibitor of androgen receptor (AR) signaling. The authors calculated a prognostic score using the Weibull multiple regression model, which assessed the correlation of ctDNA and imaging features with PFS and OS. The levels of ctDNA were significantly correlated with imaging features, e.g., SUVmax, MTV, and TLA (total lesion activity, also referred to as TLG). Patients were randomly assigned into a training (n = 68) and a test (n = 34) set. In the training set, multivariable Cox proportional hazard analyses showed that ctDNA, MTV, serum lactate dehydrogenase (LDH), and the presence of visceral metastasis were independent predictors of PFS and OS. The calculated prognostic scores were then used to group patients into three risk groups with significantly different median PFS and OS values. The parameters in the training set were then used to calculate prognostic scores in patients in the test set. The significant differences in median OS and PFS between risk groups were confirmed in the test set, indicating that combining ctDNA and imaging may improve risk stratification in castration-resistant prostate cancer.

The second study by Crispin-Ortuzar et al. showcased a machine learning framework to build a response-predictive model in patients with stage III–IV high-grade serous ovarian cancer (HGSOC) receiving NAC [34]. The framework involved integrating baseline clinical information, blood-based biomarkers (CA-125 and ctDNA), and imaging features (combined model) extracted from all primary and metastatic lesions of 92 patients. CtDNA was detected by NGS of the plasma cfDNA. The researchers then used an ensemble machine learning model that included three different algorithms: elastic net, support vector regressor, and random forest, to predict the change in total tumor volume. The model was trained using data obtained at diagnosis (training set n = 72). An internal hold-out cohort (test set, n = 20) and an independent external patient cohort (validation set, n = 42) were then used to validate the model. In the validation set, the combined model was better at predicting tumor response classification based on the RECIST guidelines (area under the receiver-operating characteristic curve, AUC = 0.8) than the clinical model (AUC = 0.5). The study showed that adding imaging data into an integrative model that included ctDNA improved the prediction of treatment response. This provides a framework for developing response-predictive models to guide NAC trials in HGSOC.

## 4. Limitations

Quantitative imaging and ctDNA biomarkers are measures of tumor burden and can be highly correlated [35]. This represents a major limitation that could negatively impact the performance of predictive models that combine these two biomarkers. Studies by the authors, however, have revealed discordances between these two measures and surmised that it is in these cases that the models could be most informative [35]. Another limitation is the lack of sensitivity of ctDNA assays, particularly in early stage cancer, when the tumor burden is low, and in the minimal residual disease setting, when the tumor has been surgically resected [45]. Previous work by the authors has also shown undetectable ctDNA levels in patients with no pathCR, indicating the limited sensitivity of the test, even in patients with extensive residual cancer burden after NAC [46,47]. New ctDNA tests with increased sensitivity are being developed to address this limitation [48]. The cost associated with testing, especially NGS-based ctDNA assays, represents another barrier that could limit widespread adoption and access to underrepresented communities. The high costs could also impede the expansion of biomarker studies to larger cohorts, which is key to building accurate models for predicting patient outcomes.

## 5. Conclusions

Our literature search yielded studies that combined imaging and ctDNA features in models predictive of treatment response and survival. The studies involved cohorts that vary in terms of the type of cancer, stage, and treatment received. Most studies used PET/CT for tumor imaging and NGS-based assays to detect ctDNA in the blood. In addition, the RECIST guidelines and PFS were often used as the response and survival endpoint, respectively.

Two studies demonstrated the feasibility of machine learning approaches for identifying predictors of outcome. Logistic regression was commonly used to predict binary outcomes (e.g., response), and Cox proportional hazard analysis was used to predict survival. Overall, the cohorts’ sample sizes were limited (40–84 patients). The authors are expanding efforts in the I-SPY2 trial to validate results from initial studies combining information from a tumor-informed ctDNA test and MRI-based FTV to build models predicting patient outcomes [1]. Since 95% of patients with a pathCR are free from metastatic recurrence after three years of follow up (i.e., 95% 3-year DRFS), the goal of I-SPY2 is to bring patients to achieve a pathCR by offering the most efficacious treatment available. Models predicting response as early as three to six weeks after treatment initiation will inform clinicians on whether to escalate or de-escalate therapy. Patients predicted to achieve a pathCR early during treatment could go to surgery early, therefore limiting exposure to the toxicity of unnecessary treatment (de-escalation). In contrast, those predicted to have a poor response may switch to a different treatment to improve the likelihood of achieving a pathCR (escalation). The authors plan to expand their studies to 1000 patients in I-SPY2, as larger training datasets are needed to create robust models to accurately predict patient outcomes. Nonetheless, despite high correlations between imaging and ctDNA features—which might imply that they lack additive value—studies in this mini-review demonstrate the feasibility of combining these biomarkers to predict treatment response and disease relapse.

## 6. Future Directions

Investigations focused on combining imaging and ctDNA features to build predictive models are in their embryonic stages. More studies are needed to determine optimal strategies for building models for predicting outcomes. This includes multimodal approaches that involve biomarkers beyond ctDNA and imaging features. For example, the I-SPY2 trial is testing whether gene expression- [49] and protein-based [50] tumor biomarkers can improve the prediction of pathCR to guide treatment selection. Wolf and colleagues in I-SPY2 have created alternative breast cancer subtypes by incorporating these molecular biomarkers into the standard breast cancer subtyping and showed improved performance in predicting treatment response [49]. The authors recently reported on the predictive and prognostic value of ctDNA in 283 patients representing two of the three major receptor breast cancer subtypes [46]. Currently, ctDNA data collection is being expanded (as discussed above) to cover all breast cancer subtypes, including patients with HER2-positive disease. Work to integrate ctDNA data with FTV using machine learning tools to identify features predictive of response (pathCR) and metastatic recurrence (DRFS) is ongoing. Adding molecular biomarkers to ctDNA and imaging information may further improve model performance. Nonetheless, larger cohorts are warranted to validate the response-predictive and prognostic models to ensure generalizability to other datasets.

## Figures and Tables

**Table 1:** Selected studies combining imaging and ctDNA biomarkers for predicting patient outcomes.

Cancer Type	Cancer Stage	Treatment	No. of Patients	Ref.
NSCLC	Stage IV	EGFR-targeted therapy	40	[36]
NSCLC	Stage I–III	Surgery, curative radiotherapy +/− chemotherapy, palliative therapy	63	[39]
BCA (luminal or ER-positive)	Stage IV	Aromatase and mTOR inhibitors	47	[38]
NSCLC	Stage III–IV	Chemotherapy	84	[37]
BCA	Stage II–III	NAC	84	[35]
PCA	Stage IV (castration-resistant)	AR signaling inhibitors	68 training set	[40]
			34 test set	
HGSOC	Stage III–IV	NAC	72 training set	[34]
			20 test set	
			42 validation set	

Abbreviations: AR, androgen receptor; BCA, breast cancer; EGFR, epidermal growth factor receptor; ER, estrogen receptor; HGSOC, high-grade serous ovarian cancer; NAC, neoadjuvant chemotherapy; NSCLC, non-small cell lung cancer. Stage I–III and Stage IV refer to early stage and metastatic settings, respectively.

**Table 2:** Imaging and ctDNA platforms used by selected studies to build models predictive of response or survival.

Imaging Modality	Imaging Features	ctDNA Assay	ctDNA Feature	Prediction Target	Statistical Model *	Ref.
CT	429 imaging (radiomic) features	NGS	Number of mutations	PFS, OS	Cox regression	[36]
^18^F-FDG PET/CT	SUVmax, MTV, TLG	Tumor-informed ddPCR or NGS	ctDNA+/−, VAF	PFS, OS	Cox regression	[39]
^18^F-FDG PET/CT	SUVmax	NGS	ctDNA+/−	PFS	Cox regression	[38]
^18^F-FDG PET/CT	SUV, MTV, TLG, IU, IC	PCR/DCE heteroduplex method	VAF	PFS, OS	Cox regression	[37]
MRI	FTV	Tumor-informed mPCR + NGS (Signatera)	ctDNA+/−, MTM/mL	pathCR, DRFS	Logistic regression, Cox regression	[35]
^18^F-FCH PET/CT	SUVmax, MTV, TLG	NGS	ctDNA fraction	PFS, OS	Cox regression, Weibull multiple regression	[40]
CT	Volume, number of lesions, disease distribution, lesion shape, texture, heterogeneity, peripheric context	NGS	TP53 VAF	Tumor volumetric response	Ensemble machine learning	[34]

Abbreviations: cfDNA, cell-free DNA; ctDNA, circulating tumor DNA; DCE, denaturing capillary electrophoresis; ddPCR, digital droplet polymerase chain reaction; DRFS, distant recurrence-free survival; ^18^F, fluorine isotope 18; CH, fluorocholine; FDG, fluorodeoxyglucose; FTV, functional tumor volume; IC, iodine concentration; IU, iodine uptake; mPCR, massively parallel polymerase chain reaction; MRI, magnetic resonance imaging; MTM, mean tumor molecules; MTV, metabolic tumor volume; NGS, next-generation sequencing; OS, overall survival; pathCR, pathologic complete response; PCR, polymerase chain reaction; PET/CT, positron emission tomography/computed tomography; PFS, progression-free survival, SUV, standard uptake value; TLG, total lesion glycolysis; VAF, variant allele frequency. * The Cox regression model is also called the Cox proportional hazards model.

**Table 3:** Endpoints, models, and summary of results from selected studies that built models predictive of response or survival.

Response Endpoint	Predictive Model	Survival Endpoint	Prognostic Model	Findings *	Ref.
n.a.	n.a.	PFS, OS	PFS, OS ~ clinical + ctDNA + imaging phenotype	1	[36]
n.a.	n.a.	PFS, OS	PFS, OS ~ ctDNA + MTV PFS, OS ~ ctDNA + TLG	2	[39]
RECIST	n.a.	PFS	PFS ~ ΔSUVmax + ctDNA (day 14)	3	[38]
RECIST	CR + PR < SD < PD ~ ∆SUVmax (%) + Follow-up ctDNA	PFS, OS	PFS ~ ΔSUVmax + follow-up ctDNA	4	[37]
pathCR	pathCR ~ FTV + ctDNA	DRFS	DRFS ~ ctDNA + FTV (+ pathCR + subtype)	5	[35]
RECIST	n.a.	PFS, OS	PFS, OS ~ MTV + ctDNA + visceral metastasis + serum LDH	6	[40]
RECIST	Tumor volume ~ clinical + CA-125 + imaging + ctDNA	n.a.	n.a.	7	[34]

* Findings: 1. Combining imaging phenotypes with ctDNA and clinical variables improved the prediction of OS and PFS. 2. ctDNA quantity was positively correlated with MTV and TLG. UniV Cox analysis showed that ctDNA detection, MTV, TLG, and SUVmax were significantly associated with PFS and OS. In a MultiV Cox model, none were associated with PFS, and only ctDNA remained a significant prognostic factor for OS. 3. The detection of ctDNA and the absence of a PET/CT response at day 14 identified patients with a low probability of benefiting from everolimus–exemestane treatment. 4. ctDNA was correlated with MTV, TLG, and IC. Follow-up ctDNA and ∆ in all PET/CT parameters were associated with survival. Adding follow-up ctDNA to ∆SUVmax improved the prediction of objective response and PFS, but not OS. 5. ctDNA provided additive value to FTV by MRI in predicting pathCR and identifying patients with reduced DRFS. 6. ctDNA, MTV, visceral metastasis, and serum LDH were independent predictors of both PFS and OS in the training set. The prognostic scores were used to group patients into three risk groups. Differences in median survival between risk groups were confirmed in the validation cohort for both OS and PFS. 7. A combined model integrating imaging and ctDNA data improved the prediction of treatment response. Abbreviations: ctDNA, circulating tumor DNA; CR, complete response; DRFS, distant recurrence-free survival; FTV, functional tumor volume; IC, iodine concentration; IU, iodine uptake; LDH, lactate dehydrogenase; MRI, magnetic resonance imaging; MTM, mean tumor molecules; MTV, metabolic tumor volume; MultiV, multivariable; n.a., not applicable; OS, overall survival; pathCR, pathologic complete response; PD, progressive disease; PET/CT, positron emission tomography/computed tomography; PFS, progression-free survival; PR, partial response; RECIST, Response Evaluation Criteria in Solid Tumors; SD, stable disease; SUV, standard uptake value; TLG, total lesion glycolysis; UniV, univariable; VAF, variant allele frequency.

## Abbreviations

The following abbreviations are used in this manuscript:

^18^F	fluorine isotope 18
AR	androgen receptor
BCA	breast cancer
cfDNA	cell-free DNA
CR	complete response
CT	computed tomography
ctDNA	circulating tumor DNA
DCE	denaturing capillary electrophoresis
ddPCR	digital droplet polymerase chain reaction
DRFS	distant recurrence-free survival
EGFR	epidermal growth factor receptor
ER	estrogen receptor
FCH	fluorocholine
FDG	fluorodeoxyglucose
FTV	functional tumor volume
HGSOC	high-grade serous ovarian cancer
IC	iodine concentration
IU	iodine uptake
LDH	lactate dehydrogenase
MAF	mutant allele frequency
mPCR	massively parallel polymerase chain reaction
MRI	magnetic resonance imaging
MTM	mean tumor molecules
MTV	metabolic tumor volume
NAC	neoadjuvant chemotherapy
NGS	next-generation sequencing
NSCLC	non-small cell lung cancer
OS	overall survival
pathCR	pathologic complete response
PCR	polymerase chain reaction
PD	progressive disease
PET	positron emission tomography
PET/CT	positron emission tomography/computed tomography
PFS	progression-free survival
PR	partial response
ptDNA	plasma tumor DNA
RECIST	Response Evaluation Criteria in Solid Tumors
ROI	region of interest
SD	stable disease
SUV	standardized uptake value
TLG	total lesion glycolysis
VAF	variant allele frequency
